# RNA Gain-of-Function in Spinocerebellar Ataxia Type 8

**DOI:** 10.1371/journal.pgen.1000600

**Published:** 2009-08-14

**Authors:** Randy S. Daughters, Daniel L. Tuttle, Wangcai Gao, Yoshio Ikeda, Melinda L. Moseley, Timothy J. Ebner, Maurice S. Swanson, Laura P. W. Ranum

**Affiliations:** 1 Department of Genetics, Cell Biology, and Development, University of Minnesota, Minneapolis, Minnesota, United States of America; 2 Institute of Human Genetics, University of Minnesota, Minneapolis, Minnesota, United States of America; 3 Department of Molecular Genetics and Microbiology, University of Florida, Gainesville, Florida, United States of America; 4 Genetics Institute, University of Florida, Gainesville, Florida, United States of America; 5 Department of Neuroscience, University of Minnesota, Minneapolis, Minnesota, United States of America; The Hospital for Sick Children and University of Toronto, Canada

## Abstract

Microsatellite expansions cause a number of dominantly-inherited neurological diseases. Expansions in coding-regions cause protein gain-of-function effects, while non-coding expansions produce toxic RNAs that alter RNA splicing activities of MBNL and CELF proteins. Bi-directional expression of the spinocerebellar ataxia type 8 (SCA8) CTG CAG expansion produces CUG expansion RNAs (CUG^exp^) from the *ATXN8OS* gene and a nearly pure polyglutamine expansion protein encoded by *ATXN8* CAG^exp^ transcripts expressed in the opposite direction. Here, we present three lines of evidence that RNA gain-of-function plays a significant role in SCA8: 1) CUG^exp^ transcripts accumulate as ribonuclear inclusions that co-localize with MBNL1 in selected neurons in the brain; 2) loss of *Mbnl1* enhances motor deficits in SCA8 mice; 3) SCA8 CUG^exp^ transcripts trigger splicing changes and increased expression of the CUGBP1-MBNL1 regulated CNS target, *GABA-A transporter 4* (*GAT4/Gabt4*). *In vivo* optical imaging studies in SCA8 mice confirm that Gabt4 upregulation is associated with the predicted loss of GABAergic inhibition within the granular cell layer. These data demonstrate that CUG^exp^ transcripts dysregulate MBNL/CELF regulated pathways in the brain and provide mechanistic insight into the CNS effects of other CUG^exp^ disorders. Moreover, our demonstration that relatively short CUG^exp^ transcripts cause RNA gain-of-function effects and the growing number of antisense transcripts recently reported in mammalian genomes suggest unrecognized toxic RNAs contribute to the pathophysiology of polyglutamine CAG CTG disorders.

## Author Summary

We describe several lines of evidence that RNA gain-of-function effects play a significant role in spinocerebellar ataxia type 8 (SCA8) and has broader implications for understanding the CNS effects of other trinucleotide expansion disorders including myotonic dystrophy type 1, Huntington disease like-2, and spinocerebellar ataxia type 7. The SCA8 mutation is bidirectionally transcribed resulting in the expression of CUG^exp^ transcripts from *ATXN8OS* and CAG^exp^ transcripts and polyglutamine protein from the overlapping *ATXN8* gene. These data suggest that SCA8 pathogenesis involves toxic gain-of-function effects at the RNA (CUG^exp^) and/or protein (PolyQ) levels. We present three lines of evidence that CUG^exp^ transcripts play a significant role in SCA8: 1) CUG^exp^ transcripts accumulate as ribonuclear inclusions that co-localize with MBNL1 in selected neurons; 2) loss of *Mbnl1* enhances motor deficits in SCA8 mice; 3) SCA8 CUG^exp^ transcripts trigger alternative splicing changes and increased expression of the CUGBP1-MBNL1 regulated CNS target, *GABA-A transporter 4* (*GAT4/Gabt4*) which is associated with the predicted loss of GABAergic inhibition within the granular cell layer in SCA8 mice. Additionally, alternative splicing changes and GAT4 upregulation are induced by CUG^exp^ but not CAG^exp^ transcripts. From a therapeutic viewpoint, it is promising that this change is reversed in cells overexpressing MBNL1.

## Introduction

Spinocerebellar ataxia type 8 (SCA8), is a slowly progressive neurodegenerative disease caused by a CTG•CAG expansion that primarily affects the cerebellum [1],[2]. The disease is transmitted in an autosomal dominant pattern with reduced penetrance and the expansion mutation was originally shown to be expressed as a CUG expansion (CUG^exp^) transcript in the 3′region of an untranslated gene, *ATXN8OS*. BAC transgenic mice expressing the SCA8 expansion (SCA8 BAC-EXP, [CTG]_116_) but not a control repeat (SCA8 BAC-CRTL, [CTG]_11_) from the endogenous human promoter develop progressive motor deficits and a loss of cerebellar GABAergic inhibition [3]. Unexpectedly, SCA8 patients and BAC-EXP mice were found to have 1C2-positive intranuclear inclusions in Purkinje cells and brainstem neurons that result from the expression of a nearly pure polyglutamine protein (ataxin 8) from a novel gene spanning the repeat in the opposite CAG direction *(ATXN8)*. The expression of CUG^exp^ transcripts from *ATXN8OS* in addition to CAG^exp^ transcripts and a polyglutamine protein from *ATXN8* suggests that SCA8 involves toxic gain-of-function effects at both the RNA (CUG^exp^) and protein (PolyQ) levels. An alternative hypothesis is that the SCA8 expansion affects the expression of an overlapping gene, *KLHL1* and although *Klhl1* knockout mice have a subtle phenotype, the relevance of this model to the human disease is unclear [4].

Substantial evidence that CUG^exp^ RNAs are toxic comes from studies of the neuromuscular disease myotonic dystrophy (DM) where CUG^exp^ (DM1) and CCUG^exp^ (DM2) RNAs alter the activities of two families of alternative splicing factors, the MBNL and CELF proteins [5],[6]. Multiple lines of evidence support the model that these expansion transcripts cause disease specific clinical features. First, transgenic mouse models of DM1 in which CUG^exp^ transcripts are expressed in the 3′ UTR of the DM1 (*DMPK*), or the unrelated *human skeletal muscle actin (HSA*), gene cause skeletal muscle myotonia and myopathy similar to human DM skeletal muscle pathology [7],[8]. In addition, CUG^exp^ or CCUG^exp^ transcripts accumulate as ribonuclear inclusions in DM1 and DM2 patient skeletal muscle [9]–[11] and alter the localization or regulation of RNA binding proteins CUGBP1 [12],[13] and MBNL1 [14],[15]. Additional studies show expression of CUG^exp^ transcripts induce alternative splicing changes in skeletal muscle genes associated with disease symptoms including the chloride channel (*CLCN1*) and insulin receptor genes (*IR*) [16]–[19]. Similar alternative splicing events in numerous genes have now been shown to be misregulated in myotonic dystrophy, often causing aberrant expression of fetal isoforms in adult tissue [5].

Evidence that MBNL1 loss-of-function plays a role in DM was suggested by studies showing MBNL1 co-localizes with CUG or CCUG ribonuclear inclusions [10] with additional strong support coming from *Mbnl1* isoform knockout mice (*Mbnl1^ΔE3/ΔE3^*) that recapitulate several aspects of the multisystemic disease pathology and misregulated splicing events characteristic of DM [20]. Furthermore, MBNL1 has been shown to bind directly to intronic elements of genes misregulated in DM cardiac and skeletal muscle (*cTNT and IR*) and to promote alternative splicing patterns normally found in adult tissue [21]. Taken together, these data suggest that expression of CUG^exp^ or CCUG^exp^ transcripts induces alternative splicing changes in DM skeletal and cardiac muscle by sequestration and functional loss of MBNL1 protein.

In addition to skeletal and cardiac muscle disease, behavioral and cognitive changes in DM suggest that CUG^exp^ and CCUG^exp^ transcripts also cause CNS effects. DM1 CUG^exp^ transcripts form ribonuclear inclusions that co-localize with MBNL1 in temporal lobe neurons of DM1 patients [22]. Additionally, alternative splicing changes in a number of CNS transcripts (*NMDA R1, APP, MAPT/Mapt, Mbnl1*) are found in humans and mice [22],[23]. Although the mechanism for these CNS splicing changes is unknown, these data suggest that DM, and possibly the much shorter SCA8 CUG expansion transcripts, cause RNA gain-of-function effects in brain.

In support of the model that SCA8 CUG^exp^ transcripts affect MBNL1 regulated pathways, expression of human SCA8 cDNA transcripts (*ATXN8OS*) in Drosophila photoreceptor neurons induce a late-onset, neurodegenerative phenotype genetically enhanced by the loss of the fly MBNL1 orthologue *muscleblind* [24]. This result is similar to fly models of DM1 [25],[26] and consistent with additional evidence for RNA gain-of-function effects in DM.

In this study, we present the first evidence that SCA8 CUG^exp^ transcripts cause “RNA gain-of-function” effects in the brain. First, we demonstrate that SCA8 CUG^exp^ transcripts form hallmark ribonuclear inclusions that co-localize with MBNL1 in humans and mice and that genetic loss of *Mbnl1* enhances motor coordination deficits in SCA8 BAC-EXP mice. Additionally, we show that expression of *ATXN8OS* CUG^exp^ transcripts dysregulate MBNL1-CUGBP1 pathways in the CNS and trigger downstream molecular changes in *GABA-A transporter 4 (Gabt4)* regulation through an RNA gain-of-function mechanism.

## Results

### Co-Localization of CUG^exp^ Ribonuclear Inclusions with MBNL1 in SCA8 Brain

**Figure 1:**
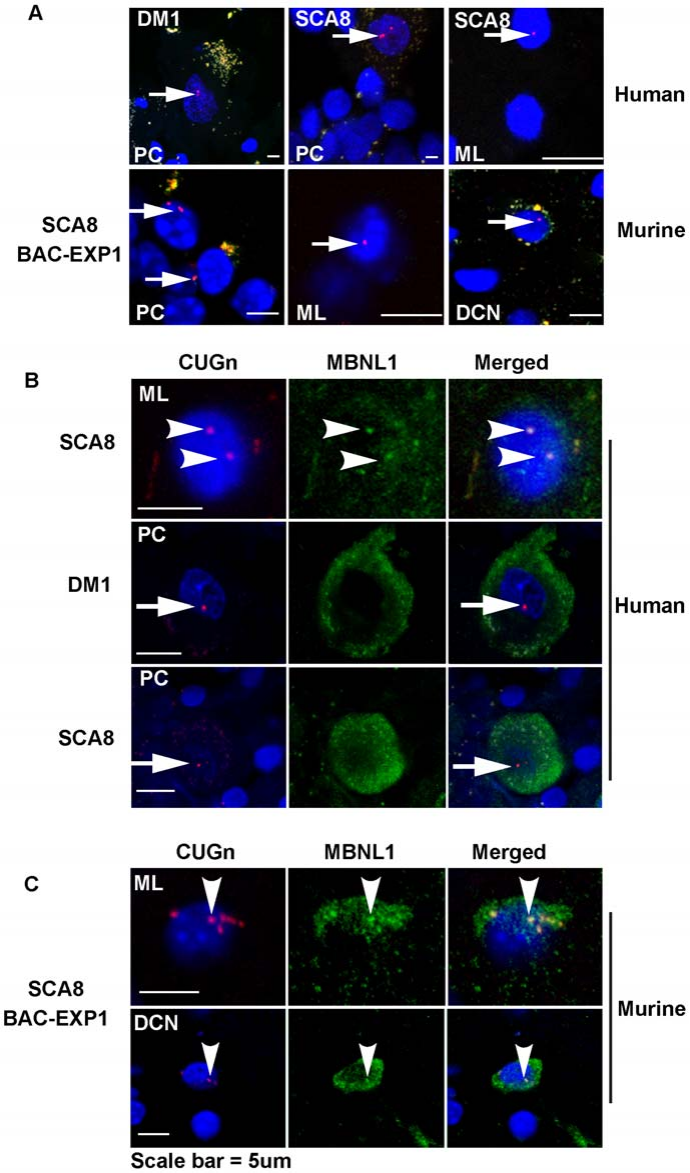
CUG^exp^–positive ribonuclear inclusions and co-localization with MBNL1 in SCA8 cerebellum. (A) CUG-positive nuclear foci (RED) in SCA8 mouse and human cerebellum. CUG ribonuclear inclusions detected by FISH (RED) in SCA8 (human and mouse) and DM1 (human) Purkinje cells (PCs), molecular layer (ML) interneurons (human and mouse), and deep cerebellar nuclei (DCN) (mouse) showing CUG positive nuclear foci (RED) in human SCA8 and DM1 cerebellum (top) and in SCA8 BAC-EXP1 mice (bottom). (B) FISH and IF analysis of CUG foci and MBNL1 in human cerebellar autopsy tissue. CUG foci co-localize with MBNL1 in molecular layer interneurons (ML) (SCA8) (top row; arrowheads) but not in Purkinje cells (PC) (SCA8 or DM1) (middle and bottom rows, arrows). Co-localization is indicated by a yellowish color when the red CUG foci, green MBNL1 and blue DAPI overlap in the nucleus. (C) CUG foci co-localize with Mbnl1 in ML interneurons and deep cerebellar nuclei (DCN) in SCA8 BAC-EXP mice (arrowheads, top and bottom rows). Images are representative examples of staining with all 3 channels acquired at equal intensities in sequential order on a confocal microscope, merged along the Z-axis, digitally zoomed, cropped, and adjusted for brightness and contrast for publication.

To determine if SCA8 *ATXN8OS* transcripts form ribonuclear inclusions similar to those found in DM, fluorescence in-situ hybridization (FISH) was preformed on SCA8 human and SCA8 BAC-EXP mouse cerebella using a Cy5-labeled (CAG)_10_ oligonucleotide probe. In human SCA8 autopsy tissue, CUG positive inclusions were found in the cerebellar cortical layers (red dots) by confocal microscopy. These CUG ribonuclear inclusions are distinguishable from background lipofuscin auto-fluorescence which appears as a yellowish-brown perinuclear staining (Figure 1A). Although ribonuclear inclusions were seen in all three SCA8 autopsy brains examined, the foci varied in distribution, size, and number between SCA8 cases and compared to ribonuclear inclusions found in DM1 cerebellum. In cerebellar tissue from SCA8 patients with 1000 and 400 CTG repeats, single CUG foci were frequently found in the nuclei of molecular layer (ML) interneurons and the Bergmann glia surrounding the Purkinje cells in the granule cell (GC) layer, while multiple smaller foci were typically found in Purkinje cells (PC). Although qRT-PCR shows CUG^exp^ transcripts are expressed at comparable levels in all three SCA8 autopsy cases, ribonuclear inclusions were not reproducibly found in the brain with 109 CTG repeats, and were only detected in a single molecular layer interneuron (not shown). While these data suggest that ribonuclear inclusions are less likely to form in patients with shorter expansions, CUG RNA foci were readily detectable in SCA8 BAC transgenic mice which express similarly sized SCA8 *ATXN8OS* (CUG)_116_ transcripts under the control of the endogenous human promoter (Figure 1A, bottom row). Similar to the RNA foci found in SCA8 autopsy tissue with 400 and 1000 repeats, the foci in these mice have a similar distribution in the cerebellar cortex (Purkinje cells, Bergmann glia, and molecular layer interneurons). Additionally, ribonuclear inclusions were found in the deep cerebellar nuclei in our mice, an area of the brain we were not able to examine in the human autopsy cases. No foci were seen in mice expressing normal repeat (CTG)_11_ or non-transgenic littermates and additionally, no CAG foci were detected using a CTG oligonucleotide probe (not shown).

FISH combined with immunofluorescence (IF) was performed to determine if the SCA8 ribonuclear inclusions co-localize with MBNL1. In human cerebellar sections, MBNL1 co-localizes with the CUG foci in molecular layer interneurons (Figure 1B). In contrast, SCA8 and DM1 Purkinje cells have clearly detectable nuclear CUG foci but these foci do not co-localize with MBNL1, which is predominantly expressed in the cytoplasm (Figure 1B, middle and bottom row arrows). Similar to the human results, SCA8 mice expressing the expansion, but not the control repeat, have CUG-positive ribonuclear inclusions that co-localize with Mbnl1 in molecular layer interneurons and deep cerebellar nuclei (Figure 1C) but not in Purkinje cells (not shown).

### Reduced Mbnl1 Enhances Rotarod Deficits in SCA8-BAC-EXP Mice

**Figure 2:**
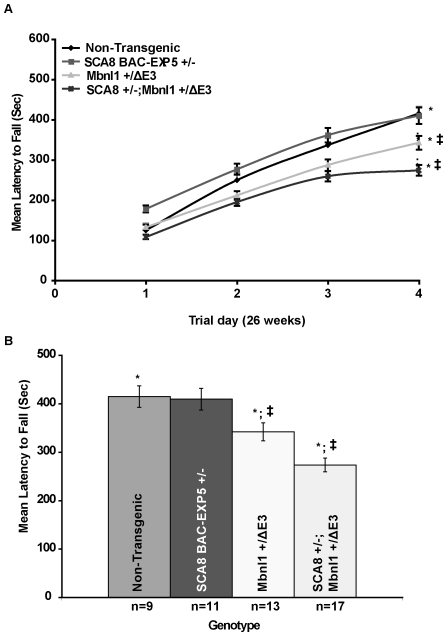
Loss of MBNL1 enhances rotarod deficits in SCA8 BAC-EXP5 mice. (A) Line plot of average latency to fall in seconds over four consecutive trial days. Individual trial day data points are the average of the last 3 trials and show a decreased latency for SCA8^+/−^; *Mbnl1* ^+/ΔE3^ mice that is significantly different from WT at day four. (B) Bar graph shows the mean latency to fall (Sec) for non-transgenic, singly transgenic SCA8 BAC-EXP5 (SCA8^+/−^). heterozygous *Mbnl1* knock-out (*Mbnl1^+^* ^/ΔE3^) and doubly transgenic SCA8 BAC-EXP5; *Mbnl1* ^+/ΔE3^ (SCA8^+/−^; *Mbnl1* ^+/ΔE3^; n = 17) littermates. As previously reported for this low-copy line (SCA8 BAC-EXP5), no significant difference between non-transgenic littermates and singly transgenic SCA8^+/−^ animals was found. Although singly transgenic *Mbnl1^+^* ^/ΔE3^ mice show a significant decrease in latency compared to non-transgenic (p = 0.01), doubly mutant SCA8^+/−^; *Mbnl1^+^* ^/ΔE3^ perform significantly worse than both WT and singly mutant *Mbnl1^+^* ^/ΔE3^ animals (p = 0.003). *and ‡ = significant differences between groups.

Because co-localization of MBNL1 with CUG^exp^ ribonuclear inclusions is thought to lead to functional impairment of nuclear MBNL1 activity in DM1, we tested the hypothesis that RNA gain-of-function effects in SCA8 contribute to the motor deficits in SCA8 BAC-EXP mice via Mbnl1 depletion. Mice from a low copy SCA8 BAC-EXP5 line (SCA8 BAC-EXP5*^+/−^* ^)^ were crossed to heterozygous Mbnl1 isoform knockout mice (*Mbnl1^+/ΔE3^*). Mice from the SCA8 BAC-EXP5*^+/−^* line were selected for these studies because these animals, which have normal rotarod performance at 26 weeks of age, do not develop a movement disorder phenotype until >1 year of age [3]. Additionally, although homozygous *Mbnl1^ΔE3/ΔE3^* knockout mice model the multisystemic features of DM pathology heterozygous *Mbnl1^+/ΔE3^* mice are similar to wild type and do not develop myotonia or other skeletal muscle changes [20]. To test if genetic *Mbnl1* loss enhances the SCA8 CNS phenotype we crossed heterozygous SCA8 BAC-EXP5*^+/−^* mice to heterozygous *Mbnl1^+/ΔE3^* knockout mice and tested the F1 offspring [(*SCA8^+/−^* (n = 11); *Mbnl1^+/ΔE3^* (n = 13); *SCA8^+/−^*; *Mbnl1^+/ΔE3^* (n = 17) and non-transgenic littermates (n = 9)] for motor deficits at 26 weeks of age by rotarod analysis. The latency to fall in seconds (sec) was recorded for 4 trials per day over 4 consecutive days. Mean differences between groups for each testing day were determined by taking the average of the last 3 trials. Consistent with previous results [3], no significant difference in mean latency to fall was found between the SCA8 BAC-EXP5*^+/−^* mice (409.52±22.51 sec) and non-transgenic littermates (414.83±22.18 Sec.; P = 0.86). Although heterozygous *Mbnl1^+/ΔE3^* mice did have a significantly different mean latency to fall (342.15±18.75 sec.) compared to non-transgenic littermates [F(1, 86) = 6.22; P = 0.012], double mutant offspring (S*CA8^+/−^*; *Mbnl1^+/ΔE3^*) performed significantly worse (273.69±14.13 Sec) than either the singly mutant *Mbnl1^+/ΔE3^* (P = 0.003) or *SCA8* BAC-EXP5*^+/−^* littermates [F(1,102) = 31.27; P<0.00001] (Figure 2). These data provide genetic evidence that loss of Mbnl1 plays a role in SCA8 pathogenesis and support the hypothesis that SCA8 CUG^exp^ transcripts affect Mbnl1 regulated pathways in the brain.

### Identification of Candidate RNA Gain-of-Function Targets by Cross-Linking and Immunoprecipitation (CLIP)

**Figure 3:**
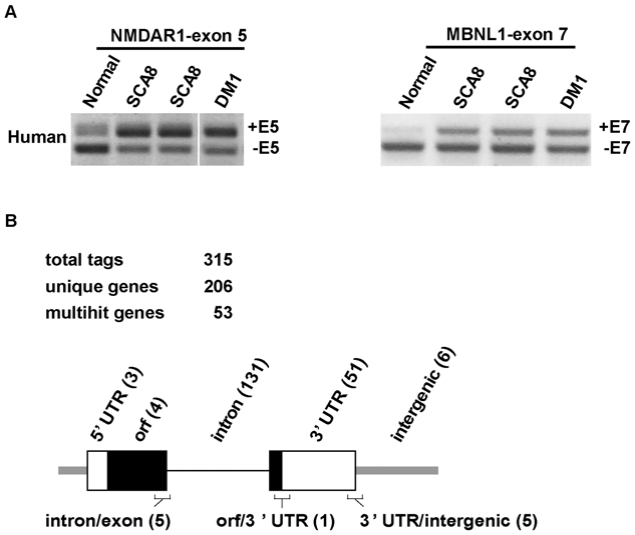
Alternative splicing changes in SCA8 and CUGBP1 RNA targets. (A) RT-PCR of human autopsy tissue showing alternative splicing changes previously reported for DM1, NMDAR1 (exon 5) and MBNL1 (exon 7) also occur in SCA8. B) Diagram summarizing relative position of CUG-BP1 CLIP tags in genes. Boxes = exons (open = UTR; black-ORF); thick grey line = intergenic regions. Also indicated are tags that overlap intron/exon, orf/3′ UTR and 3′ UTR/intergenic junctions with the number of unique tags for each location in parentheses.

The discovery that CUG^exp^ RNA and MBNL1 protein co-accumulate in ribonuclear foci in neurons suggests that, similar to the dysregulation of MBNL/CELF pathways in DM muscle, the accumulation of SCA8 CUG expansion transcripts might lead to the dysregulation of developmentally regulated splicing patterns in SCA8 brain. Further support for this hypothesis comes from alternative splicing changes found in MBNL1 and NMDAR1 in SCA8 autopsy brains (Figure 3A) which mimic previously reported changes in DM1 [22],[27]. To identify novel splicing targets that might be affected by Mbnl1 loss or increased Cugbp1 activity we used cross-linking and immunoprecipitation (CLIP) analysis [28] on mouse postnatal day 8 (P8) hindbrains. CLIP was successful in identifing RNA targets of Cugbp1 but not Mbnl1. Because CUG-BP1 and MBNL1 have been shown to be antagonistic regulators of alternative splicing of a number of different targets from work done in the myotonic dystrophy field, we reasoned that CUG-BP1 CLIP tags would be also be good candidate targets for MBNL1 regulation. At P8, hindbrain can be readily dissociated into a cell suspension which was then exposed to UV-light to fix RNA-protein complexes formed *in vivo*. This procedure avoids artifacts associated with immunopurification of unfixed proteins which may redistribute during *in vitro* manipulation. Cross-linked RNA-protein complexes were treated with RNase T1, to generate relatively short (60–200 nt) CLIP RNA tags, and immunopurified with a monoclonal antibody against Cugbp1. Complexes were then subjected to electrophoresis/electroblotting and filter-retained RNA tags were identified following cDNA conversion, amplification and DNA sequencing. A total of 315 Cugbp1-associated RNA tags were identified representing 206 genes with 53 multi-hit tags (Figure 3B and Table S1). While the majority of these tags were intronic (64%), in agreement with a previous study on the splicing regulator Nova1 [28], a significant number (25%) were positioned within 3′ untranslated regions (UTRs). Sequence analysis revealed that Cugbp1 CLIP tags were enriched in UG repeats consistent with previous three-hybrid and SELEX studies which have indicated that the highest affinity sites for CUGBP1, CUGBP2 and CELF4 are (UG)_n_, and (UGUU)_n_ repeats [29],[30]. A recent study identified a GU-rich 11-mer element (GRE) UG(U)_3_G(U)_3_GU, which is enriched in the 3′-UTRs of short-lived transcripts, as a CUGBP1 binding motif [31]. Interestingly, only one Cugbp1 3′ UTR CLIP tag (Slc22a5), and two intronic tags (Lrrc9, Samd12), contained this GRE motif (see Table S1).

From this list of potential Cugbp1 targets we focused on the *gamma-aminobutyric acid (GABA-A) transporter 4* gene, *Gabt4*, because previous *in vivo* functional imaging studies showed a loss of cerebellar GABAergic inhibition in our SCA8-BAC-EXP mice [3]. Further analysis of the *Gabt4* CLIP tag showed that the putative Cugbp1 binding site maps to exon 7 (and overlaps the exon 7 5′ splice site) which is highly conserved in human and mouse. The gene name in mouse is *Gabt4/Slc6a11* and in human *GAT3* but for clarity referred to here as *Gabt4* and *GABT4*, respectively.

### Upregulation of GABA-A Transporter 4 (Gabt4) and Functional Granular Cell Layer Changes in SCA8 Mice

**Figure 4:**
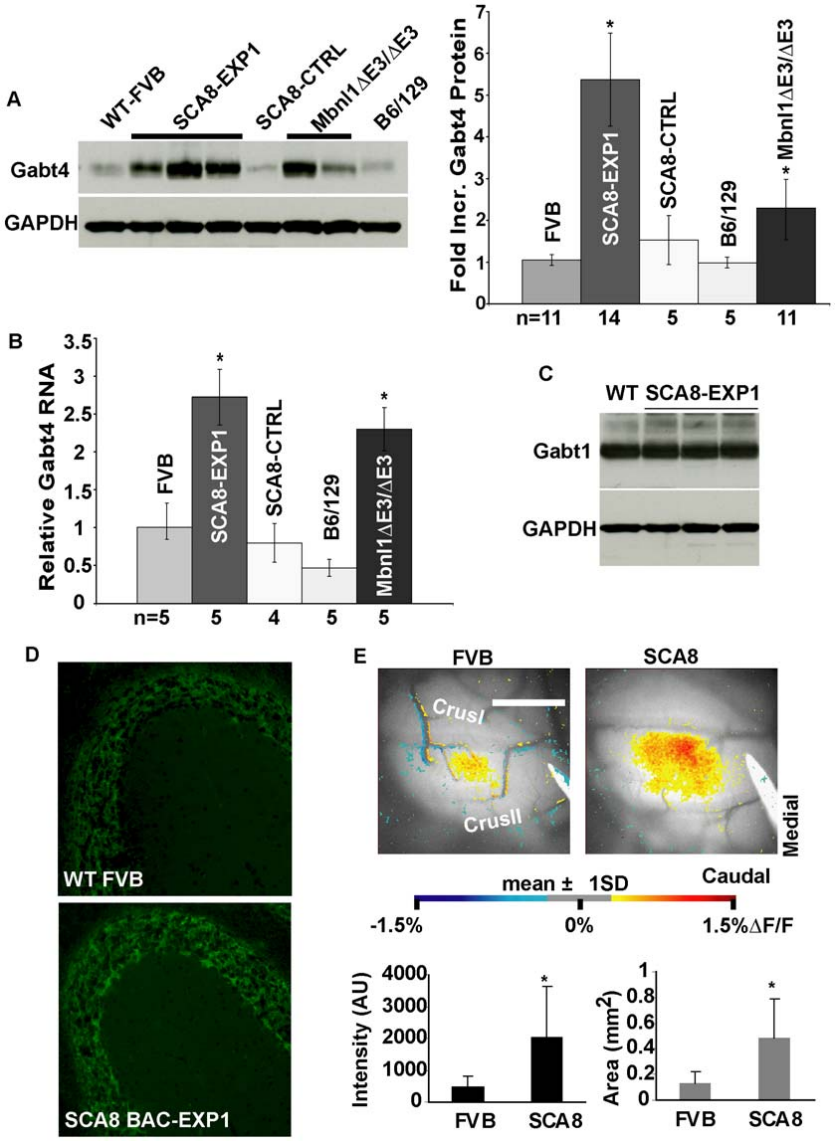
Upregulation of *Gabt4* in SCA8 BAC-EXP1 and *Mbnl1* ^ΔE3/ΔE3^ mice. (A) Example immunoblot showing increase in Gabt4 protein in SCA8 BAC-EXP1 and Mbnl1^ΔE3/ΔE3^ mice relative to strain specific WT controls. Bar graph shows average Gabt4 protein quantified by densitometry methods, and analyzed for mean between group differences compared to strain specific wild type animals. Asterisks = significant differences for SCA8 (p<0.002) and *Mbnl1^ΔE3/ΔE3^* (p<0.001) mice compared to non-transgenic controls. (B) qRT-PCR showing increased *Gabt4* vs. *Hprt* in SCA8 BAC-EXP and *Mbnl1* ^ΔE3/ΔE3^ compared to BAC-CTRL and non-transgenic littermates. Asterisks = statistical significance, p = 0.0015 for SCA8 and p<0.01 for *Mbnl1* ^ΔE3/ΔE3^ mice compared to non-transgenic controls. (C) Example protein blot showing no change in another prominent cerebellar GABA transporter receptor, Gabt1, in SCA8 BAC-EXP1 mice. (D) Fluorescent IHC staining showing a similar distribution with a qualitative increase in Gabt4 IF in cerebellar granular cell layer in SCA8 BAC-EXP1 compared to wildtype mice. (E) Optical responses in Crus II to stimulation of the ipsilateral C3 whisker pad in FVB and SCA8 mice showing increased cerebellar cortical response to whisker pad stimulation in SCA8 mice. Shown are the thresholded (mean±1 SD of the control region) and pseudocolored responses superimposed on an image of the background fluorescence. Scale bar = 1 mm. Both the intensity and area of the responses were greater in the SCA8 mice compared with FVB control mice. *denotes p<0.05.

Since *Gabt4* was identified as a putative target of CUGBP1 by CLIP analysis and because increased *Gabt4* expression could explain the increased cortical loss of GABAergic inhibition previously reported in our mice by reducing GABA at the synapse, we hypothesized that SCA8 CUG^exp^ RNA dysregulates Cugbp1 and Mbnl1 pathways resulting in an increase in Gabt4 expression. Consistent with this idea, we found significant increases in cerebellar Gabt4 protein and RNA levels by protein blot (5.36±1.11 fold; p = 0.003) and qRT-PCR (2.72±0.68 fold p = 0.0015) in SCA8 BAC-EXP1 mice compared to non-transgenic littermates (Figure 4A and 4B) while no increase was seen in SCA8 BAC-CTRL animals (Figure 4B). Further supporting the hypothesis that Gabt4 increases in SCA8 are caused by sequestration of Mbnl1 by SCA8 CUG^exp^ transcripts, Gabt4 protein and transcript levels are also higher in *Mbnl1^ΔE3/ΔE3^* knockout mice compared to strain matched (129/B6) non-transgenic littermate controls with a 2.49±0.083 (P<0.001) and 2.29±0.32 (P = 0.013) mean fold increase by protein blot and qRT-PCR, respectively (Figure 4A and 4B). The increases in Gabt4 protein were reproducible in both high copy number SCA8 BAC-EXP lines studied (BAC-EXP1 and BAC-EXP2) but more variable in mice from the lower copy number BAC-EXP5 line (data not shown) consistent with the decreased penetrance reported previously in this line. In addition, no similar changes were seen in Gabt1 levels, another member of GABA-A transporter family expressed in cerebellum indicating that changes seen in *Gabt4* are not caused by a general upregulation of GABA-A transporters (Figure 4C).

Immunofluorescence studies show expression of Gabt4 protein is primarily localized to the granular cell layer of the cerebellar cortex and the deep-cerebellar nuclei (DCN) and that no overt change in this distribution is seen (Figure 4D) between wildtype and SCA8 BAC-EXP1 mice. To further characterize the loss of inhibition phenotype and the possible role of Gabt4 we examined the SCA8 BAC mice for functional changes in the granular layer of the cerebellum, the site of highest Gabt4 expression using flavoprotein optical imaging in response to whisker pad stimulation in SCA8 mice [32],[33]. Activation of cerebellar granule cells by mossy fibers is in part controlled by Golgi cell mediated feedback that produces GABAergic inhibition of granule cells [34]. Because the clearance of synaptically released GABA in the granular layer is predominately mediated by Gabt4, up-regulation of Gabt4 would be expected to reduce this Golgi mediated inhibition and enhance the responses to mossy fiber input. The imaging data collected confirm this prediction. Whisker pad stimulation evokes a patch-like response (Figure 4E) consistent with previous electrophysiological and imaging studies [32],[35]. There is a significant increase in both the intensity and area of the response in Crus II in the SCA8 BAC-Exp (n = 5) compared with FVB mice (n = 7) (Figure 4, p<0.05). Therefore, up-regulation of Gabt4 in the granular layer is associated with the expected increase in the response to mossy fiber inputs activated by peripheral stimulation.

In summary, these data demonstrate that *Gabt4*, a gene identified by CLIP analysis as a Cugbp1 target, is upregulated at the RNA and protein levels in both our SCA8 BAC-EXP1 and *Mbnl1^ΔE3/ΔE3^* knockout animals but not in the SCA8 BAC-CTRL mice expressing a normal length CUG_11_ repeat. Additionally, Gabt4 upregulation in these mice is associated with the predicted loss of GABAergic inhibition within the granular cell layer.

### GABT4 Upregulation and Alternative Splicing in SCA8 Human Autopsy Tissue

**Figure 5:**
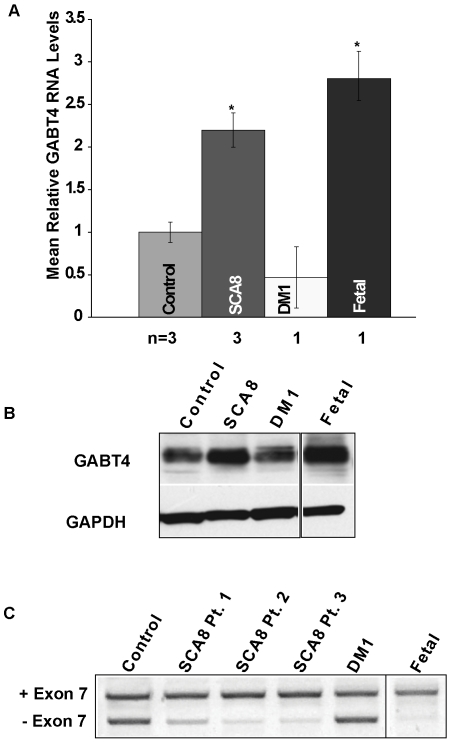
Upregulation and alternative splicing of *GABT4* in human SCA8 brain. (A) qRT-PCR shows increased GABT4 RNA in frontal lobe autopsy tissue from adult SCA8 (n = 3) and control fetal (n = 1) tissue. * = significant difference (p<0.01). (B) Protein blot showing up-regulation of GABT4 in SCA8 and fetal frontal lobe vs. DM1 and Control with GAPDH as loading control. (C) RT-PCR of GABT4 shows a shift favoring exon 7 inclusion transcripts in SCA8 and control fetal frontal lobe tissue vs. adult control and DM1.

To determine if *GABT4* upregulation also occurs in humans, we examined steady-state RNA and protein levels in human SCA8 autopsy brains. While expression of the SCA8 CUG^exp^ and CAG^exp^ transcripts and GABT4 overlap in both the cerebellum and the frontal lobe, only frontal lobe tissue was suitable for analysis because of significant cell loss in the cerebellum caused by neurodegeneration in SCA8 patients. Total RNA extracted from adult SCA8, DM1 and control and from 26-week fetal frontal cortex was examined by qRT-PCR using primers to exons 1 and 2. SCA8 autopsy brains (n = 3) showed increased *GABT4* transcripts levels compared to adult control (p<0.01) (Figure 5A) and similar levels to those found in fetal tissue. A similar trend of increased GABT4 protein was also seen in SCA8 and fetal brain compared to adult control or DM1 tissue (Figure 5B).

Because exon 7 of *Gabt4* was identified as a potential Cugbp1 binding site, we investigated if increases in *GABT4* expression could be related to misregulation of exon 7 alternative splicing. Consistent with this hypothesis, all three human SCA8 autopsy brains showed a shift in alternative splicing favoring inclusion of *GABT4* exon 7 containing transcripts compared to adult control (Figure 5C). Preferential exon 7 inclusion was also seen in control human fetal autopsy tissue but not in adult DM1 tissue and is correlated with the increases in GABT4 RNA and protein seen in SCA8 adult and control fetal brain (Figure 5). Interestingly, no exon 7 splicing shifts or increases in *GABT4* expression were found in DM1 autopsy tissue, possibly reflecting differences in spatial or temporal expression of the SCA8 and DM1 CUG^exp^ transcripts.

Similar to the dysregulation of other genes in DM, SCA8 CUG^exp^ transcripts trigger a shift in the ratios of alternatively spliced human *GABT4* (+/− exon 7) transcripts that resemble those found during fetal development. Sequence analysis shows that transcripts skipping exon 7 would lead to the introduction of a premature termination codon (PTC) which would be predicted to target (−) exon 7 transcripts for nonsense mediated decay (NMD). These data suggest a model in which developmental alternative splicing changes in human normally lead to lower levels of GABT4 in adult tissue and higher levels in SCA8 and during fetal development.

Similar to humans, Gabt4 is also up-regulated in the SCA8 BAC-Exp and *Mbnl1* ^ΔE3/ΔE3^ mice. Although alternative splicing of exon 7 has not yet been detected in the mouse by RT-PCR, exclusion of mouse exon 7 would also create a PTC (in exon 10) that would be predicted to lead to NMD. Further studies are needed to determine if NMD in the mouse leads to more efficient degradation of (−) exon 7 transcripts which prevent their detection or if Gabt4 upregulation in the mouse occurs via another mechanism.

### SCA8 CUG^exp^ Not CAG^exp^ Transcripts Misregulate GABT4 Pre–mRNA Alternative Splicing and Expression in SK-N-SH Cells

**Figure 6:**
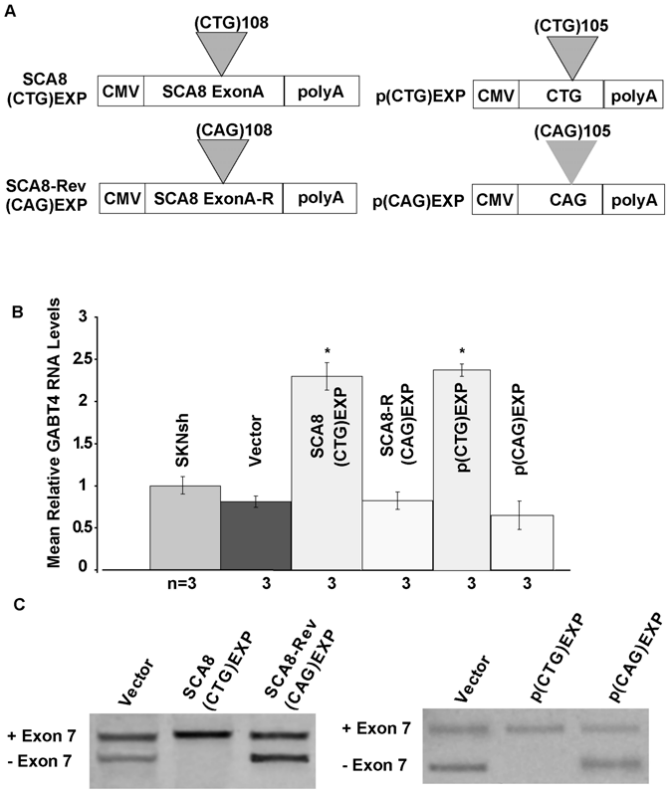
Endogenous *GABT4* up-regulation in SK-N-SH cells induced by CUG^exp^ but not CAG^exp^ transcripts. (A) Schematic of constructs used to express SCA8 CUG^exp^ or CAG^exp^ and pure CUG^exp^ and CAG^exp^ transcripts. (B) qRT-PCR shows increased GABT4 RNA levels in SK-N-SH cells expressing Exon A CUG^exp^ or pure polyCUG^exp^ transcripts. * = statistical significance, p<0.0001. (C) Alternative splicing shifts favoring exon 7 inclusion in cells expressing SCA8 Exon A CUG^exp^ or pure polyCUG^exp^ that does not occur in response to SCA8 Exon A CAG or pure polyCAG^exp^ transcripts compared to vector alone.

To test directly if increases in *GABT4* are induced by SCA8 CUG^exp^ or CAG^exp^ transcripts, we examined their effects in human neuroblastoma SK-N-SH cells. Transient transfections were performed using minigenes (Figure 6A) designed to express SCA8 CUG (SCA8-CTG^exp^) or CAG expansion transcripts (SCA8-REV CAG^exp^). Cells expressing SCA8 CUG^exp^ transcripts show significant increases in *GABT4* RNA levels by qRT-PCR relative to untransfected cells (p = 0.007) or to cells transfected with vector alone (p = 0.006) while expression of the SCA8 CAG^exp^ construct compared to vector alone had no effect (p = 0.72) (Figure 6B). Additionally, transient transfections of minigenes without *ATXN8* and *ATXN8OS* flanking sequence show poly-CUG_105_ but not poly-CAG_105_ transcripts up-regulate *GABT4* RNA (p = 0.001) (Figure 6C). Further analysis using primers flanking exon 7, show cells expressing higher levels *GABT4* transcripts also preferentially express higher ratios of exon 7 included transcripts. (Figure 6B and 6C).

### MBNL1 Overexpression Reverses SCA8 CUG^exp^–Induced Misregulation of *GABT4* in SK-N-SH Cells

**Figure 7:**
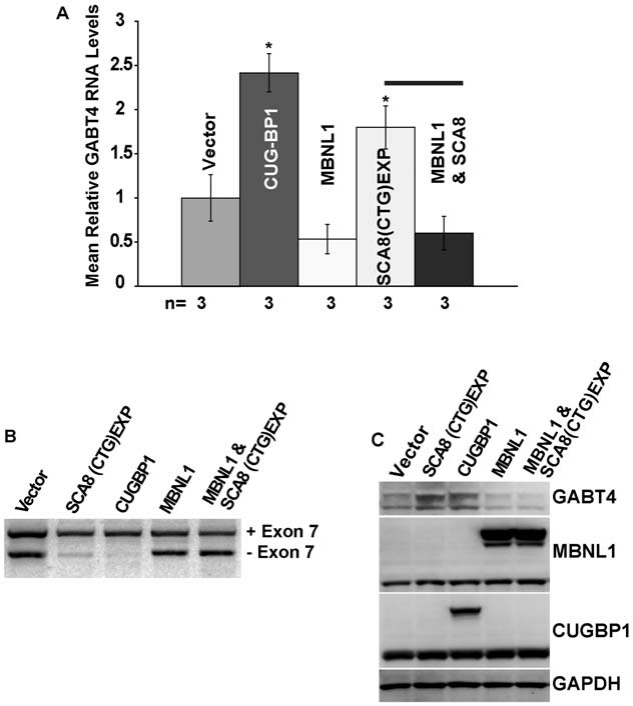
Antagonistic Regulation of GABT4 by CUGBP1 and MBNL1 in SK-N-SH cells. (A,B) qRT-PCR showing increased GABT4 transcript levels and alternative splicing changes favoring exon 7 inclusion in cells overexpressing CUG^exp^ transcripts or CUG-BP1 but not in cells overexpressing MBNL1 1/41 or CUG^exp^ and MBNL1 1/41. (C) Protein blot showing GABT4 is increased in cells overexpressing CUG^exp^ transcripts or CUG-BP1, but not in cells overexpressing MBNL1/41 alone or MBNL1/41 and CUG^exp^ transcripts.

To test if *GABT4* expression is antagonistically regulated by CUGBP1 and MBNL1, we transfected SK-N-SH cells using GFP-tagged human MBNL1/41 and CUGBP1 minigenes capable of inducing alternative splicing changes in cell culture [21],[36]. As above, alternatively-spliced exon 7 transcripts were assayed by RT-PCR with primers located in *GABT4* exons 6 and 8 and primers spanning exons 1 and 2 were used to assess endogenous levels of *GABT4* by qRT-PCR. Cells overexpressing CUGBP1 (p<0.0001) or SCA8 exon A CTG^exp^ minigenes show increases in *GABT4* RNA (p = 0.003) and a concomitant increases in protein and a splicing shift favoring exon 7 inclusion compared to vector alone (Figure 7A–7C). While no change in GABT4 RNA or protein was seen in cells overexpressing MBNL1/41 alone, overexpression of MBNL1/41 and SCA8 CUG^exp^ transcripts reverses the increase in GABT4 RNA (p<0.0001) and protein (Figure 7A and 7C) triggered by SCA8 CUG^exp^ transcripts alone and restores exon 7 alternative splicing ratios to the normal adult pattern (Figure 7B). Sequence analysis of the *GABT4* RT-PCR products confirm the upper and lower bands include and exclude exon 7 respectively and that the (-) exon 7 transcripts have a premature stop codon in exon 8. Taken together, these results are consistent with a model in which SCA8 CUG^exp^ transcripts alter the regulation of *GABT4* by sequestration of MBNL1 and/or an increase in the expression or activity of CUGBP1.

## Discussion

The SCA8 CTG CAG mutation is bidirectionally expressed and produces both CUG and CAG-expansion transcripts and a nearly pure polyglutamine expansion protein [3]. We investigated RNA gain-of-function effects in SCA8 and present three lines of evidence that CUG^exp^ transcripts play a role in SCA8. First, we demonstrate CUG^exp^ transcripts accumulate as ribonuclear inclusions in selected cells and that these RNA foci co-localize with Mbnl1 in a subset of neurons in SCA8 patients and mice. Second, we show genetic loss of *Mbnl1* enhances motor coordination deficits in low-copy SCA8 BAC^exp^ mice. Third, we demonstrate SCA8 CUG^exp^ transcripts trigger increased expression of a CUGBP1-MBNL1 regulated CNS target, *GABT4*, in both mice and humans as well as in a human cell culture model and demonstrate the predicted loss of GABAergic inhibition within the granular cell layer occurs in these animals.

Although CNS effects are a clinically important feature of myotonic dystrophy, mechanistic studies have focused on skeletal and cardiac muscle and little is known about the effects of CUG^exp^ transcripts in brain. In addition, the antagonistic relationship between CUGBP1 and MBNL1, previously documented in heart and skeletal muscle, has not been demonstrated in the CNS. In this study we used CLIP analysis to identify putative Cugbp1 CNS targets. To explore the molecular basis of the loss of GABAergic inhibition we investigated changes in expression levels and alternative splicing of the Cugbp1 CLIP target, *Gabt4*, in our mice. We show that overexpression of CUGBP1 in human SK-N-SH cells, or depletion of MBNL1, result in an upregulation of *GABT4* that mimics the *in vivo* changes in steady state levels caused by CUG^exp^ transcripts. These data provide the first evidence that CUG^exp^ RNA gain-of-function effects in the brain involve the dysregulation of CUGBP1-MBNL1 pathways. These data also suggest that MBNL1 overexpression, which has been demonstrated to be therapeutic in skeletal muscle, might also be an effective treatment to reverse pathological changes associated with expression of CUG^exp^ transcripts in the CNS. Additionally, the long list of other putative Cugbp1 targets identified in mouse brain provides an important future resource for the identification of additional CNS genes dysregulated in SCA8 and DM.

Previous studies in myotonic dystrophy suggest that the expression of CUG^exp^ transcripts, together with MBNL1 and CELF proteins and their downstream target genes need to be coordinated temporally and spatially for disease pathogenesis. Therefore, defining the temporal and cell specific *ATXN8OS* expression pattern will be crucial for understanding disease pathogenesis and interpreting future results. We identified for the first time in SCA8, CUG-RNA foci in Purkinje cells, molecular layer interneurons and the deep cerebellar nuclei. In addition, we show MBNL1/Mbnl1 colocalizes with SCA8 CUG foci in molecular layer interneurons and the DCN. Interestingly, although Purkinje cells had nuclear CUG foci in both DM1 and SCA8, co-localization with nuclear MBNL1 was not observed in these cells. Further studies will need to be done to determine if MBNL1 is expressed in the nucleus of these cells or if changes in MBNL1 splice forms affect its ability to bind to the CUG expansion transcripts. Taken together, these results suggest that temporal and spatial expression patterns of expansion transcripts and the overlap in expression of specific RNA binding proteins and their downstream target genes are likely to underlie the susceptibility of specific cells to RNA gain-of-function effects and the clinical difference between DM and SCA8.

To investigate the broader significance of MBNL1 in SCA8, we tested the effects of Mbnl1 depletion on a behavioral phenotype in our SCA8 mice by crossing heterozygous *Mbnl1^+/ΔE3^* animals with a low-copy SCA8-BAC-EXP5 line. The phenotypic enhancement of the rotarod deficits found in the doubly transgenic animals with reduced Mbnl1 suggests that expression of CUG^exp^ transcripts in SCA8 and the subsequent downstream effects on Mbnl1 are sufficiently significant to contribute to the movement disorder phenotype found in SCA8.

*GABT4* upregulation in human SCA8 autopsy tissue and cell culture studies supports a model in which *GABT4* levels are regulated by alternative splicing changes and the NMD pathway. Similar to the dysregulation of other genes in DM1 (*CLCN-1*, *IR*) [5], the expression of CUG^exp^ but not CAG^exp^ transcripts [37] triggers a shift in the ratios of alternatively spliced *GABT4* transcripts that resembles those found during fetal development.

Further studies are needed to determine if *GABT4* alternative splicing changes occur throughout the brain and to directly show if alternative splicing of exon 7 and NMD regulate *GABT4/Gabt4* expression levels in both humans and mice. Additional studies are also required to directly test the hypothesis that *GABT4* upregulation causes the decrease in GABAergic inhibition seen in our SCA8 BAC-EXP mice.

While these data provide the first evidence for RNA gain-of-function effects in SCA8, it is also possible that the polyQ expansion protein contributes to the disease. In contrast to other disorders in which polyQ expansions are expressed as part of a mature protein, the SCA8 CAG^exp^ is expressed as a nearly pure polyQ tract. While previous studies have shown that pure polyQ repeats are toxic in Drosophila and mice, the relative contribution of RNA and protein gain-of-function effects in SCA8 still needs to be assessed.

In this study we provide several lines of evidence that RNA gain-of-function effects play a significant role in SCA8 and show that SCA8 CUG^exp^ transcripts affect alternative splicing patterns controlled by MBNL1 and CUGBP1 in the mammalian brain. While SCA8 is the first reported disease in which a single expansion mutation expresses both a polyglutamine protein and CUG^exp^ transcripts, bidirectional expression has also been recently described in other triplet expansion disorders. For example, sense (CUG^exp^) and antisense (CAG^exp^) transcripts have been reported at the DM1 locus [38]. Similarly, Huntington disease Like 2 (HDL2), another disease in which CUG^exp^ transcripts form ribonuclear inclusions [39], also has 1C2-positive inclusions (1C2 is an antibody that recognizes polyglutamine expansions), suggesting bidirectional expression may also occur in that disease [40],[41]. Additionally, bidirectional expression across the FMR1 CCG CGG repeat in Fragile X tremor ataxia patients has also been reported [42]. Our data showing that relatively short (∼110 repeats) CUG^exp^ transcripts can cause dysregulation of MBNL1/CUGBP1 regulated pathways and the growing number of antisense transcripts recently reported in mammalian genomes [43], highlight the need to look for CUG transcripts expressed at other loci traditionally associated with polyglutamine expansion disorders.

## Materials and Methods

### FISH and Immunofluorescence

Fluorescent in-situ hybridization (FISH) and immunofluorescence (IF) was performed on frozen parasagittal cerebellar sections (6 µm) as described [10]. Sections were fixed in 4% paraformaldehyde for 30, permeabilized in 2% acetone for 5′, incubated in 30% formamide/2XSSC pre-hybridization for 1 hr at RT and hybridized with a Cy5-(CAG)_10_ for 2 hr at 42^°^C and post-hybridized at 45^°^C for 30′. Sections were coverslipped and stained with DAPI to identify CUG RNA foci or incubated overnight at 4^°^C with Mbnl1 antibody (polyclonal A2764 gift of C.A. Thornton) at 1∶1000 and visualized by Alexa 488 secondary antibody (1∶2000) at RT for 30′. A2764 is a polyclonal antibody, directed against a C-terminal peptide of Mbnl1, **s**pecificity was demonstrated by lack of reactivity on immunoblots from Mbnl1^ΔE3/ΔE3^ mice and the antibody recognizes all known alternative splice isoforms of Mbnl1 [27]. Co-localization of Cy5-(CAG)_10_ RNA foci with MBNL1 was done by confocal microscopy (Olympus, Fluoview 1000) in 3 fluorescent channels with ≥5 layers (0.5 µm) compressed along the Z-axis and then merged. Gabt4 immunofluorescence staining was done using 10 µm frozen sections fixed using 4% paraformaldehyde for 30′, permeabilized with 2% acetone for 5′, briefly washed with PBS and then blocked in 5% goat serum, 0.3% Triton X-100 in PBS for 2 hr at 4°C. Sections were incubated with anti-Gabt4 polyclonal antibody (human GAT3, Sigma, St. Louis, MO) diluted 1∶1000 in the same blocking solution for 24 hr at 4°C, washed in PBS four times for 20′ and incubated with secondary antibody (Alexa 488 goat anti mouse IgG, Molecular Probes, Eugene, OR) using a 1∶2000 dilution for 2 hr at RT. Sections were washed 5 times for 5′ each in PBS and DAPI counterstained and coverslipped using Vectastain with DAPI (Vectashield, Ca)

### Rotarod Analysis

Rotarod training was performed at 26 weeks of age using an accelerating rotarod (Ugo Basille, Comerio, Italy) as described [3]. All mice tested were F1 littermate progeny of single copy integrant SCA8 BAC-EXP5^+/−^ and *Mbnl1^+/ΔE3^* mice. Four trials were run per day for four days: averages of the four trials on day four are presented. rmANOVA followed by post-hoc analysis (Tukey's HSD) was performed to assess differences in rotarod performance between groups (SCA8 BAC-EXP5^+/−^; *Mbnl1^+/ΔE3^*; SCA8 BAC-EXP5^+/−^/*Mbnl1^+/ΔE3^* and non-transgenic littermates).

### Northern Analysis

For RNA analysis, 15 µg of total RNA isolated from non-transgenic FVB or Bl6/129 wild type, SCA8 BAC-EXP1, SCA8 BAC-CTRL and *Mbnl1* ^ΔE3/ΔE3^ cerebellum was separated on a Northern Max-Gly glyoxal gel (Ambion), transferred to a nitrocellulose membrane, cross-linked by ultraviolet radiation and hybridized at 65^°^C in Rapid-Hyb buffer (Amersham) using a [^32^P]dUTP *in vitro* transcribed RNA probe to nucleotides 576–996 (NM_172890.3) of the mouse *Gabt4* gene. Expression analysis was performed relative GAPDH by densitometry and analyzed by one-way ANOVA with Tukey's HSD post-hoc comparisons when necessary.

### Quantitative RT–PCR

Two step qRT-PCR was performed on an ABI Prism 7500 Real Time PCR System (Applied Biosystems, Foster City, CA). Total RNA was isolated from mouse cerebellum, human frontal lobe autopsy tissue or transiently transfected SK-N-SH cells. cDNA was generated from 5 µg of total RNA using 1^st^ Strand Synthesis Supermix primed with random hexamers (Invitrogen). Relative qRT-PCR was performed on 1 µl of cDNA with qRT-PCR SYBER Green Master Mix UDG with ROX (Invitrogen) using mouse specific primers (RSD1013 5′- CCT CTG AAG GCA TCA AGT TCT ATC TGT ACC-3′) (RSD1014 5′-GTT GTT GTA ACT CCC CAG AGC GGT TAG-3′) or human specific primers for autopsy and SK-N-SH cells (RSD1009 5′- AAC AAG GTG GAG TTC GTG CT-3′) (RSD1010 5′- ACT TGT GAA CTG CCC CAG AG-3′). Two stage PCR was performed for 40 cycles (95^°^C – 15″, 60^°^C– 1′) in an optical 96 well plate with each sample cDNA/primer pair done in triplicate. Relative quantification compared to strain specific control was estimated using the threshold cycle (Ct) of GABT4/Gabt4 normalized to the Ct of the housekeeping gene *Hprt* or *GAPDH* for mouse and human, respectively. Dissociation curve analysis and ethidium bromide gel analysis was used to assess PCR product purity at the end of each qRT-PCR run. Statistical analysis was done using rmANOVA on the mean normalized Ct value of the 3 trials per sample and compared by Tukey's HSD post-hoc analysis for differences between groups when necessary.

### Immunoblotting

Animals were sacrificed and half of the cerebellum was rinsed with PBS and lysed in 450 µl of RIPA buffer (150 mM NaC1, 1% sodium deoxycholate, 1% Triton X-100, 50 mM Tris-HC1 pH 7.5, 100 ug/ml PMSF) for 45′ on ice. Cell lysates were centrifuged at 16,000×g for 15′ at 4 °C and the supernatant was collected. 20 µg of protein were separated on a 10% NuPAGE Bis-Tris gel (Invitrogen), transferred to nitrocellulose membrane (Amersham), blocked in 5% dry milk in PBS containing 0.05% Tween 20 and probed with an anti-GAT4 antibody (human GAT3; Sigma; 1∶1000) or anti-GAT1 antibody (ABcam; 1∶1,000) in blocking solution and then incubated with anti-rabbit or anti-mouse HRP conjugated secondary antibody (Amersham). Mean fold increase in protein levels were determined by densitometry normalized to GAPDH and compared to non-transgenic littermates for each blot. Statistical significance was determined by one-way ANOVA with between group comparisons evaluated by Tukey's HSD when necessary.

### cDNA Constructs

ExonA of containing the SCA8 expansion was amplified by PCR from the BAC transgene construct, BAC-exp, using the 5′ primer (5′CGAACCAAGCTTATCCCAATTCCTTGGCTAGACCC-3′) containing an added *HindIII* restriction site and the 3′ primer(5′ACCTGCTCTAGATAAATTCTTAAGTAAGAGATAAGC-3′) containing an added *XbaI* restriction site. This *HindIII/XbaI* fragment was cloned into the pcDNA3.1/*myc*-His A vector (Invitrogen, CA). The *SCA8* ExonA cDNA was placed under the control of the CMV promoter of plasmid pcDNA3.1/*myc*-His. To construct ExonA-Rev, the *HindIII/XbaI* fragment of *SCA8* ExonA was subcloned in the reverse orientation into pcDNA3.1/myc-His vector. The pCTG^exp^ and pCAG^exp^ clones (108 repeats) were generated by PCR amplification of SCA8 Exon A with added EcoRI restriction sites. This EcoRI/EcoRI fragment was cloned into the pcDNA3.1/myc-His A vector. The integrity of all constructs was confirmed by sequencing. Both pEGFP-N1-CUGBP1 and pEGFP-C1-MBNL1/41 have been described [27],[44],[45].

### Cell Culture and Transfections

SK-N-SH cells were cultured in DMEM medium with 10% fetal bovine serum at 37°C with 5% CO_2_. Transient transfections were performed using 1 µg of SCA8 repeat expressing plasmids, pEGFP-N1-CUGBP1, or pEGFP-C1-MBNL1/41 minigenes and Lipofectamine 2000 Reagent (Invitrogen, Carlsbad, CA). Cells were collected 48 hrs post-transfection for expression analysis.

### Alternative Splicing

Analysis of NMDAR1 exon 5 and MBNL1 exon 7 alternative splicing were conducted using total RNA collected from SCA8, DM1 and control human brain autopsy tissue using Trizol (Invitrogen, CA) reagent according to the manufactures procedures. Human NMDAR1 exon 5 splicing was determined by amplification of exon 4-5-6 using PCR primers hsGRIN1 ex4 For 5′- GCGTGTGGTTTGAGATGATG -3′; hsGRIN1 ex6 Rev5′-GGTCAAACTGCAGCACCTTC -3′. Similarly, exon 7 alternative splicing of human MBNL1 was determined by amplification of exon 6-7-8 using PCR primers hsMBNL1 ex6 For 5′-GCTGCCCAATACCAGGTCAAC -3′; hsMBNL1 ex8 Rev 5′-TGGTGGGAGAAATGCTGTATGC -3′. Determination of GABT4 exon 7 alternative splicing was done by reverse transcribing 2 µg total RNA from frontal lobe or SKN-S-H cells as described above. 10% of this reaction was subjected to PCR using primers (RSD1004 5′-GTTGTATACGTGACTGCGACATT-3′; RSD1011 5′-GTTCAGGCAACAGAGCATGA-3′) to amplify nucleotides 791 – 1057 of human GAT3 (NM_014229.1) for 25 cycles at 94^°^C-45″, 54^°^C-30″, 72^°^C-1′ followed by 72^°^C for 6′. PCR products were run out on a 1.5% agarose gel with ethidium bromide and bands containing exons 6-7-8 (267 bp) or 6–8 (163 bp) were cut and verified by sequencing.

### Identification of Cugbp1-Associated Transcripts

The crosslinking and immunoprecipitation protocol (CLIP) was performed as described [28],[46] with minor modifications. Hindbrains were dissected from mouse postnatal day 8 (P8) C57BL/6J pups followed by dissociation in 1X Hank's balanced salt solution containing 10 mM HEPES, pH 7.3 and UV-irradiated to crosslink RNA-protein complexes. Cells were lysed and RNA was partially digested with RNase T1 to produce 30–200 nt fragments. Lysates were cleared by ultracentrifugation and Cugbp1-bound fragments were immunoprecipitated using mAb 3B1 and Protein G Dynabeads (Invitrogen, Carlsbad, CA). Following 3′-end addition of RNA linkers, 5′ ends were labeled with g^32^P-ATP, protein-RNA complexes were eluted, separated by electrophoresis and protein-RNA complexes transferred to nitrocellulose. Bands corresponding to 60–70 kDa (10–20 kDa larger than the 50 kDa Cugbp1 protein) were excised from the nitrocellulose and the RNAs released by proteinase K digestion. RNA fragments were size fractionated by denaturing PAGE followed by 5′-end RNA linker ligation, RT-PCR and DNA sequencing.

### Animal Preparation and Flavoprotein Optical Imaging

All animal experimentation was approved by and conducted in conformity with the Institutional Animal Care and Use Committee of the University of Minnesota. Experimental details on the animal preparation, optical imaging and stimulation techniques are only briefly described as these have been provided in previous publications [32],[33]. Mice (3–8 months old) were anesthetized with urethane (2.0 mg/g body weight), mechanically ventilated, and body temperature feedback-regulated. The electrocardiogram was monitored to assess the depth of anesthesia. Crus I and II of the cerebellar cortex were exposed and the dura removed. An acrylic chamber was constructed around the exposed folia and superfused with normal Ringer's solution.

The animal was placed in the stereotaxic frame on an X-Y stage mounted on a modified Nikon epifluorescence microscope fitted with a 4×objective and a 100 W mercury-xenon lamp. Images of Crus I and II were acquired with a Quantix cooled charge coupled device camera with 12 bit digitization (Roper Scientific). The images were binned (2×2) to 256×256 pixels with a resolution of ∼10 µm×10 µm per pixel. Flavoprotein autofluorescence was monitored using a band pass excitation filter (455±35 nm), an extended reflectance dichroic mirror (500 nm), and a>515 nm long pass emission filter [33].

To evoke peripheral responses, the ipsilateral 3C vibrissal pad was stimulated with a bipolar electrode (tips ∼1 mm apart) using 20 V, 300 µs pulses at 10 Hz for 10 s [32]. Parallel fiber stimulation was performed throughout the experiment to test the general physiological condition of the cerebellar cortex. To activate parallel fibers and their postsynaptic targets (Purkinje cells and interneurons), an epoxylite-coated tungsten microelectrode (∼5 M) was placed just into the molecular layer [33].

The basic imaging paradigm consists of collecting a series of 10 control frames (background) followed by a series of 500 experimental frames with an exposure time of 200 ms for each frame (Metamorph Imaging System, Universal Imaging Corp.). Whisker pad stimulation was initiated at the onset of the experimental frames. This was repeated 4 times and the 4 series were averaged. Each pixel in this series of average images was converted into the change in fluorescence above background (ΔF/F) [32]. The maximal response to whisker stimulation occurred in frames 31–75 and these frames were averaged to generate the response image. A pixel was defined as responding to the peripheral stimulation by the following threshold procedure. First, the response image was low-pass filtered (3×3) and the mean and standard deviation (SD) of the pixels in a control region (usually a corner of the image) were determined. The pixels above the mean + 1 SD of the control region were considered to respond to the stimulus. The response area was defined as total area of all the pixels responding and the response intensity as the sum of the DF/F of all responding pixels. Differences in the area and intensity of the responses between the SCA8 and FVB mice were evaluated using a Student's *t*-test (α = 0.05). For display the pixels above or below this mean±1 SD were pseudo-colored and superimposed on an image of the folia studied.

## Supporting Information

Table S1 Candidate RNA Gain-of-Function Targets by Cross-Linking and Immunoprecipitation (CLIP).
(0.18 MB XLS)
